# A Relational Approach to the Design for Peer Support

**DOI:** 10.3390/ijerph18052596

**Published:** 2021-03-05

**Authors:** Yoonyee Pahk, Joon Sang Baek

**Affiliations:** 1 School of Design and Human Engineering, UNIST, UNIST-gil 50, Ulju-gun, Ulsan 44919, Korea; iyoonee@unist.ac.kr; 2 Department of Human Environment and Design, Yonsei University, Yonsei-ro 50, Seodaemun-gu, Seoul 03722, Korea

**Keywords:** aging, social network, social support, peer-support, relational, design

## Abstract

Peer-support services enhance mental wellbeing and increase the knowledge and capabilities of self-help groups in various settings. To ensure that these services foster peer-support relationships as intended, it is necessary to design and assess them from a relational perspective. This study presents a relational framework for peer-support design and its application to two existing peer-support services for solitary seniors in Seoul and Ulsan. The framework aims to support the analysis and conception of peer-support services. It incorporates network analysis and codesigning to understand multi-faceted peer-support relationships and to develop strategies for creating relational values, respectively. Case studies used observation and interviews to understand the multi-faceted issue of social support. Relational data for fourteen solitary seniors were collected and analyzed in terms of the qualities, quantities, and structure of peer-support relationships. Analysis results demonstrate an increased level of perceived peer support through relationship forming, as well as the factors that suppress peer-support building such as network fragmentation and the discrepancy of needs. Analysis results were fed into codesigning interventions with stakeholders. Based on these findings, we discuss the preconditions for building peer-support relationships and outline the relational approach to the design for peer support in a wider context.

## 1. Introduction

### 1.1. Peer Support and Aging

Peer or mutual support occurs when people provide help to their peers. It is based on networks that enable people to exchange support [1], and empower them as co-producers of support, rather than passive beneficiaries [2]. Existing studies report that peer support enhances mental wellbeing and increases the knowledge and capabilities of self-help groups in various settings [3,4,5]. Peer support also contributes to social inclusion and the empowerment of seniors by engaging them in activities aimed to meet their own needs as well as those of others [6].

South Korea is experiencing an unprecedented surge in its aging population, and one out of five seniors live alone [7,8]. Solitary seniors are prone to social isolation, loneliness, and depression [9,10]. Both the government and civil sector have responded by introducing peer-support programs at the community and national levels over the last decade. One such example is Colourful Youth, developed by the Yangcheon-gu District in Seoul. This program supports men in their 50s who are living alone and characterized as both independent and reluctant to receive support even if they are socially, emotionally, and economically vulnerable. As the number of lonely deaths, i.e., dying alone at home and remaining unnoticed for some time, among these individuals has steadily increased (accounting for 35.8% of all lonely deaths in South Korea [11]), a group of social enterprises have initiated a peer-support service to provide them with emotional support. The service organizes weekly meetings that consist of various group activities, including board games, story sharing, and peer mentoring. Colourful Youth is thus an intervention designed to foster peer support for individuals with a condition for which support networks do not typically emerge spontaneously.

There are several limitations in the way existing studies approach developing peer-support services. Firstly, most support the adaption of existing services with operational guides and toolkits rather than new service development. While the former is beneficial for outlining stepwise and evidence-based processes, it also limits design creativity. Secondly, existing toolkits are used to evaluate the outputs and outcomes of peer-support services solely within the functional (or technical) dimension (i.e., how the perceived level of peer support has changed due to an intervention) and tends to neglect the relational (or social) dimension (i.e., how the peer-support network has changed due to an intervention). This study was motivated to fill these gaps and poses the following questions: (1) How do we understand the relational dimension of peer-support services? (2) What needs to be considered in order to design favorable conditions for peer support to develop? (3) How do we design peer-support services from a relational perspective? We looked for answers by devising an analytic framework, applying it to real-world cases of peer-support services for solitary seniors and proposing a relational approach to the design for peer support.

### 1.2. Designing for Peer Support

Support relationships are dynamic, transient, and uncontrollable [12]. In service design, this has helped create the perspective that relationships comprise an infrastructure for services with a subject that is to be designed for rather than to be designed [13,14]. One can thus design for relational qualities such as peer support by deliberately shaping the environment or conditions in which interpersonal encounters are formed and facilitated. Cipolla [15] reported a need for methodologies that support designers in understanding and dealing with interpersonal interactions that are highly personal and dynamic, whereas Baek et al. [16] argued that existing studies have tended to focus on how the technical dimension of the service (e.g., service concepts, processes, and interfaces) is designed. There has thus been less focus on the social dimension (e.g., the relational characteristics and qualities among service users).

Previous peer-support studies have come from a variety of disciplines, including health services, biomedical informatics, medicine, engineering, and information and communication. These research efforts have typically addressed specific diseases or living conditions (e.g., mental illnesses, diabetes, HIV, cancer, university campuses, and rural communities). However, few studies have explored peer-support design, and they are limited in number and scope, consisting mainly of guidelines, case studies, and toolkits. Theoretical studies have analyzed the behavioral and environmental characteristics of peer supporters and provided guides for social-support interventions. For instance, The Australian Centre for Social Innovation (TACSI) [17] reported that the type and quality of interpersonal connections influence quality-of-life for seniors. These results supported the idea that a framework was needed to assess and design peer-support services. Weiss and colleagues [18] developed six recommendations for the design, implementation, and evaluation of online social support programs for cancer survivors. Case studies described peer-support networks involving varied themes and forms and have provided related datasets for building theories and methodologies [17,18,19]. Other studies introduced toolkits with step-by-step operational guides for implementing peer-support services in various settings—for instance, helping community members and health professionals establish and maintain cancer-support groups [20], outlining the development of peer support in communities or universities [21], providing comprehensive guidelines for delivering peer support services (including backstage operations involved in preparing organizational cultures to better adopt peer support or recruit and hire peer staff) [22], and conducting action-based research to develop university peer programs [19].

### 1.3. Measuring Social (or Peer) Support

**Figure 1:**
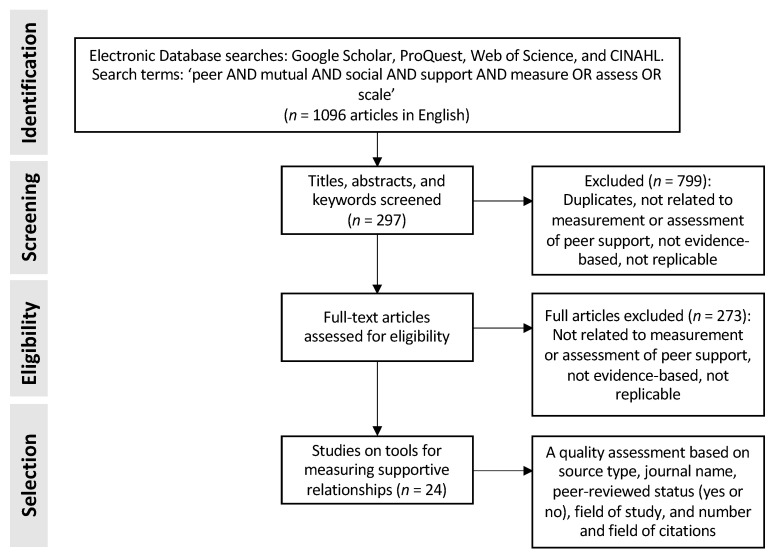
Systematic literature review process.

We searched for different ways to assess peer-support relationships by conducting a systematic literature review of studies on peer support measurement. The initial results were limited, and we expanded our search boundary to the measurement of social support (Figure 1). This was done because social support is a broader concept, representing the ‘exchange of resources between at least two individuals’ [23]. Here, exchanges are not necessarily equivalent, but are mutating and foster a movement towards shared goals [19]. The review protocol consisted of the following steps: (1) search for and identify literature using search terms, (2) read the title, abstract, and keywords to determine study relevance, and (3) read full articles when relevant or skim publications for relevant chapters to determine whether the article should be included or extracted based on the selection criteria [24]. The search terms were ‘peer AND mutual AND social AND support AND measure OR assess OR scale’. Searches in four databases (i.e., Google Scholar, ProQuest, Web of Science, and CINAHL) returned 1096 articles. These were further reduced to 297 articles by excluding duplicates, those that were not related to measurement or assessment of peer support, and those that were not evidence-based or not replicable (step 2–3). After step 3, we identified 24 tools for measuring supportive relationships. A quality assessment was then conducted based on the following criteria: source type, journal name, peer-reviewed status (yes or no), field of study, and number and field of citations (ibid.).

Literature studies revealed that most studies analyze the functional dimension of support relationships. Some studies suggested that a deeper understanding of support networks could be achieved by analyzing the relational dimension along with the functional [25,26,27,28]. For instance, analysis of the functional dimension reveals the properties and functions associated with social support (e.g., emotional, informational, and tangible support) [29], whereas the relational dimension offers insights into the structural characteristics of the network [26]. House et al. (ibid) argued that social support is manifested in three different but interconnected aspects of social relationships: quantity, structure, and quality. Certain support-relationship quantities (e.g., the number, strength, and density of support relations) are necessary conditions for both the structure and qualities of the relationship. The structure and qualities influence each other because the former is a manifestation of the latter. House et al. (ibid.) also identified the network characteristics related to social support, such as size, density, reciprocity, multiplexity, durability, intensity, frequency, dispersion, and homogeneity. More recent studies have used metrics such as network size, tie strength, density, centrality and centralization, cliques, reciprocity, and multiplexity to assess social support [25,28,30].

## 2. Methods

### 2.1. Case Studies

Case studies were conducted to understand the peer-support networks of solitary seniors in South Korea and conceive interventions to reinforce these networks. The case studies involved a collaboration with two organizations that provide peer-support services for solitary seniors in South Korea. They were selected by: (1) conducting literature and case studies of peer-support services for solitary seniors in South Korea, (2) identifying potential partners (e.g., social welfare centers and social enterprises), and (3) contacting the identified organizations and reviewing their qualification based on their motivations and accessibility.

**Figure 2:**
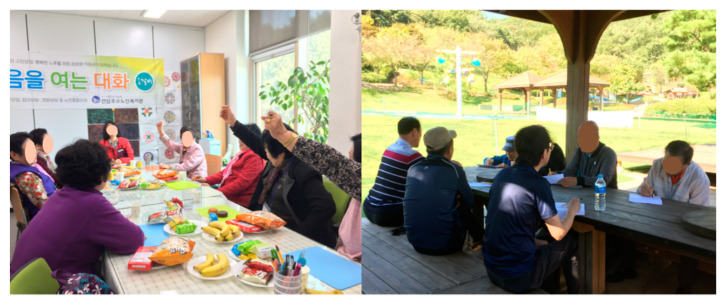
Group activities in Befriending (**left**) and Colourful Youth (**right**).

The first case is Befriending, which was organized by a senior welfare center in Ulsan. Here, participants consisted of seven solitary seniors suffering from social isolation and/or depression. As the name suggests, the service aimed to help lonely seniors make friends with one another. Participants met once each week to perform various activities (e.g., expressing their emotions and conditions through fine art, and relaying thoughts and personal stories). Soon after the program began, the program manager encountered situations in which participants conflicted with one another and dropped out of program. This made her wonder what hindered their active engagement. The other case was Colourful Youth, as introduced earlier. In both cases, program managers wanted to know how effective their service was and how they could improve it to better foster supportive relationships (Figure 2).

**Figure 3:**
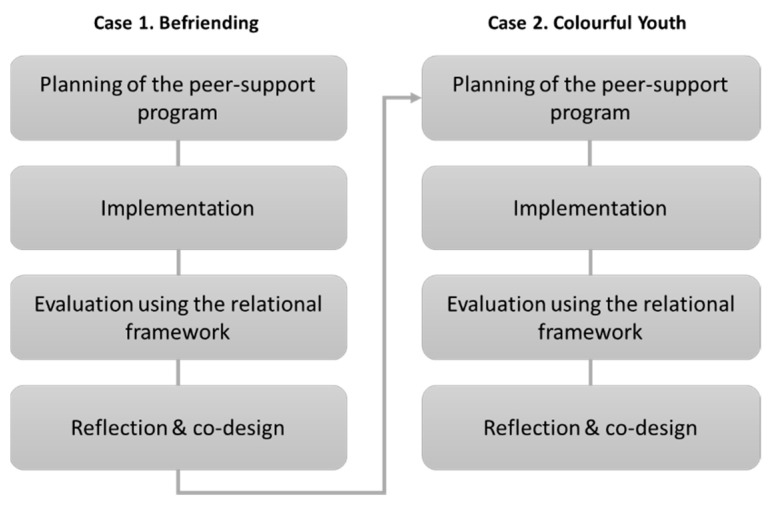
The case study process.

Each study followed the process of planning, implementing, evaluating, and reflecting and codesigning a peer-support service (Figure 3). In order to observe users regularly and in close proximity, the first author participated as a staff member in the planning and implementation of both cases. She also applied the analytical tools and facilitated the codesign sessions.

### 2.2. Data Collection

The data collection process employed mixed methods to understand the multi-faceted issue of social support [25]. We used observation and interviews to collect both qualitative and quantitative data to aid in our understanding of the qualities, quantities, and structural composition of social relationships (e.g., how mutually supportive a social network is and how it is structured).

Program participants were recruited by the partnering organizations. In Befriending, the welfare center approached solitary seniors who lived in low-income areas, who had survived or were vulnerable to suicidal behaviours, and/or who had been diagnosed with loneliness or depression. Seven people were recruited—all female, aged between 65 to 79—and participated for thirteen weeks from March to June, 2017. In Colourful Youth, the program targeted a cohort vulnerable to lonely death. The district office identified them by conducting a full investigation of socially isolated and economically vulnerable males in their 50s, and invited them to the program. Seven people were recruited and participated for fifteen weeks from July to November, 2017.

We collected data using observation and interviews during and after the implementation, respectively. We collected data following the protocols of the American Psychological Association (APA) Ethical Principles and Code of Conduct [30]. We presented to the program participants the purpose of the study, its procedure and duration, anticipated benefits, and the data protection policy. Written informed consent was obtained from those who agreed to participate in the research for recording images and voices. All data were pseudo-anonymized.

We collected quantitative data such as the type of peer support, level of perceived support, and name of support providers based on the Norbeck Social Support Questionnaire (NSSQ) [31], which is one of the most recognized tools for assessing social-support relationships according to the systematic literature review (Figure 1). NSSQ was developed by Norbeck and colleagues to measure the perceived strength of social support. It consists of nine questions on a five-point Likert scale; the first six collect the functional content of support relations (i.e., affective support, affirmative support, and aid), whereas the rest measure the duration, frequency, and absence of relations. Here, affective support is defined as a situation where one expresses admiration, respect, liking, and/or love towards another, thereby providing the recipient with the notion of care [32,33,34]. Affirmative support is an agreement or acknowledgment of the appropriateness of some act or statement concerning another person’s behaviors, perceptions, beliefs, or expressed views [32,35]. Aid refers to tangible, instrumental, and/or informational support (e.g., advice or feedback) [36] or direct aid (e.g., taking care of someone) [34]. The reliability of NSSQ has been tested through test-retest comparisons and intercorrelations among all items [37].

We revised the NSSQ by adding questions 7–12, associated with peer-support network quality. These questions aimed to collect information on the facilitators and barriers associated with building support relationships, participant needs, and design opportunities (Table 1). We also modified our data collection methods to make it more user-friendly. Althoughthe original study relied on surveys, we conducted face-to-face interviews at participants’ homes. The questionnaire was rephrased more intuitively and the response scale was designed with pictorial icons after learning that some seniors had difficulty remembering names and/or reading the text. Interviews were semi-structured and held individually with all fourteen participants. Each interview lasted between 20–40 min, and was recorded and transcribed.

### 2.3. Data Analysis

**Figure 4:**
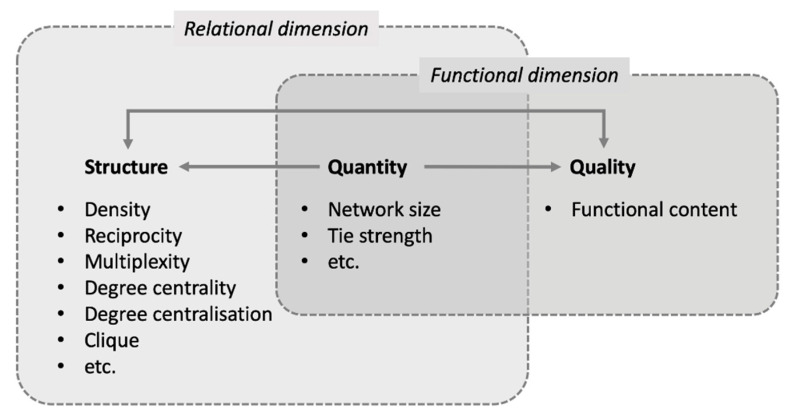
Theoretical model for analyzing peer-support networks.

Once the data collection was complete, we analyzed and visualized peer-support networks using UCINET 6 and Gephi, and analyzed the qualitative data obtained from observations and interviews using content analysis. For the peer-support network analysis, a theoretical model to guide the analysis was developed based on the literature. We paid particular attention to the contexts in which earlier studies measured support networks and the metrics they used [25,26,27,28,38]. In this model, examinations of network structure yield an understanding of the relational dimension of peer support and examinations of network quality lead to an understanding of the functional dimension, although network quantity is related to both the relational and functional dimensions (Figure 4).

We then selected the metrics for measuring a support network. The selection process included reviewing the relevance and applicability of the metrics to the case under examination, investigating how these metrics had been used in previous studies, as well as internal discussions. The selected metrics for measuring structure were density, reciprocity, degree centrality, and clique. These are useful for interpreting the structural meaning of social grouping (or isolation) and the significance of an individual during communication. For measuring quantity, we selected network size and tie strength, all of which relate to the amount of support present in social relationships. We chose functional content of support relationships as the metric to measure quality (Table 2).

The qualitative data were analyzed using content analysis. Two researchers designed the coding framework based on the typology of social support used in NSSQ, i.e., affective support, affirmative support, and aid [31]. Among other typologies of social support, we chose it for consistency with the questionnaire. Each researcher coded the complete dataset and compared the results to identify disagreements, which were then discussed, and refinements were made until all disagreements were resolved. The inter-rater reliability of the coding frame was validated using Krippendorff’s alpha value (Kalpha). The test result was reasonably reliable with Kalpha = 0.812 [41]. We then triangulated our findings, which involved comparing metric values with verbal and behavioral data and examining consistency for each participant. Interpretation of the results led to both problems and opportunities for reinforcing peer support.

Analysis results were fed into codesigning interventions with program managers and participants. We organized a codesign workshop with the stakeholders from each case to promote collective creativity for brainstorming interventions to improve user retention and reinforce peer-support construction [42]. The participants included three practitioners from Befriending and two practitioners and three users from Colourful Youth. Each workshop was semi-structured and ran for 60–90 min in the following order: (1) introducing the workshop; (2) reporting analysis results, including the peer-support network analysis and the participant need analysis; (3) identifying any other issues and problems concerning peer-support relationships; and (4) discussing the results and brainstorming strategies to reinforce peer support. The research team moderated discussions and facilitated idea generation. The data were recorded, transcribed, and analyzed for triangulation with the quantitative data.

## 3. Results

### 3.1. Befriending

#### 3.1.1. Network Quantity

In this study, the tie strength of an interpersonal relationship is defined as the perceived intensity of peer support. The tie strength of each support type is estimated from the values of corresponding questions in the questionnaire (Table 2). Tie strengths within Befriending varied in support type, with the highest being affective support (1.70), followed by aid (1.21) and affirmative support (0.95). These results aligned with the service goal of fostering affective support.

#### 3.1.2. Network Quality

During the interviews, we asked the participants to explain what helped and impeded them in building peer support by describing the functional content of their support relationships. In Befriending, the affective support exchange was hampered by a few members’ bad manners and careless behaviors. Affirmative support building was impeded by a lack of trust among peers, privacy intrusions, low self-esteem, and network fragmentation. Although the participants looked for aid, including information about the programs and events in the senior center, only a few of them exchanged such aid within their cliques. In the following section, we elaborate and interpret these issues in relation to network structure.

#### 3.1.3. Network Structure

**Figure 5:**
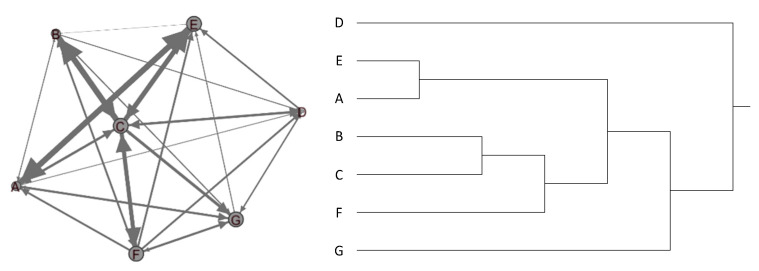
A sociodiagram (**left**) and hierarchical clustering (**right**) of the Befriending program.

The Befriending peer-support group consisted of seven female seniors. During the five-month program, most participants developed support relationships in this small group, thus forming a densely connected group (density = 0.95). Degree centrality varied among participants of the Befriending program. Participant C exhibited the highest level, whereas D exhibited the lowest, suggesting an actively engaged member and potential outliers in the group. The analysis of the clique demonstrated that participants A, B, and E were closer to each another than D and G (Figure 5). We found the extent of group fragmentation harmful to some participants, who felt isolated and unsupported. D exhibited low self-esteem and was hesitant to tell her stories to the group. She and B expressed dismay to see that the group had become divided. G complained that A and C often dominated conversations with boastful statements (e.g., bragging about their children), which made her uncomfortable. G eventually left the program and later told us that the program failed to meet her expectation of making intimate friends.

#### 3.1.4. Intervention Ideas

During the workshop, the practitioners acknowledged that the current approach to team building was problematic and needed to change. This involved considering participant needs and expectations of the service by finding common and cohesive denominators to overcome differences in age, income, and education level, which all acted as barriers to forming peer support. They thus proposed a session to understand participant motivations and expectations on the first day of the program, which then feeds into group formation. They also decided to reform ground rules so that participants would be more aware of privacy issues, while maintaining respect for others. The new rules would be co-created with the participants to increase their acceptance and empower them to reflect on the consequences of their behaviors. These new ground rules would also promote participant autonomy and further clarify the responsibility of nurturing and preserving peer support. In addition, the practitioners brainstormed strategies to strengthen relationships among distant participants such as rapport-building, celebrating memorable days, and playing games that facilitated the communication of feelings (e.g., the emotion cards used in psychotherapy settings). Another barrier to relationship building was low self-esteem, which prevented seniors from being prosocial. The corresponding idea was to encourage participants to give positive oral feedback or write similar messages to one another.

### 3.2. Colourful Youth

#### 3.2.1. Network Quantity

In Colourful Youth, the tie strength of affirmative support was the highest (1.79) followed by affective support (1.60) and aid (1.37). It is noteworthy that, overall, participants wanted to receive aid more than affective support which the program aimed to provide, i.e., tangible, instrumental, and informational aid to lessen the economic hardship during unemployment. As the program progressed, the gap between what it could offer and what (some of) the participants demanded became increasingly evident.

#### 3.2.2. Network Quality

There was a clear difference in perceived levels of affective support between participants who were in cliques and those who were not. The former tended to perceive more affective support than the latter, indicating that network fragmentation is associated with peer-support building. Empirical data have shown that fragmentation amplifies peer-support building within cliques and aggravates it outside of cliques. We also observed that the exchange of peer support spontaneously developed into cliques. In the beginning, the participants found it difficult to open their minds to strangers, even though they acknowledged the importance of it in building peer support. It was one person’s courage to be honest about himself that broke the ice: B and C felt deeply empathetic toward and intimate with D when D displayed the courage to open his mind and tearfully described his unhappy past. D also played a central role in providing aid in the group. He was well informed about how to get financial support during unemployment and frequently shared information on job opportunities and welfare benefits with B, C, F, and G. Participants needed considerable aid: information about financial support was in particular high demand. D was knowledgable about these issues and frequently shared information on job opportunities and welfare benefits with B, C, F, and G.

#### 3.2.3. Network Structure

**Figure 6:**
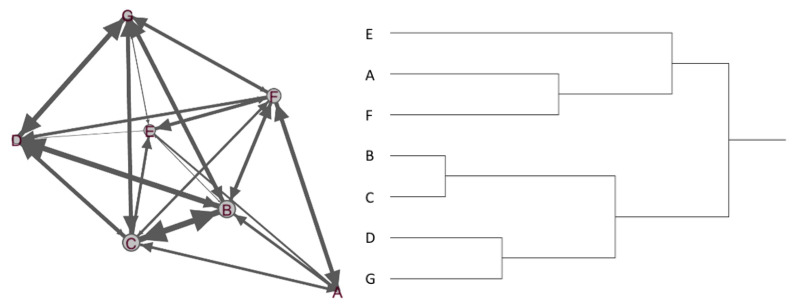
A sociogram (**left**) and hierarchical clustering (**right**) for the Colourful Youth program.

Network fragmentation developed over time in the Colourful Youth program. The result was rather polarizing—during the three-month program, B, C, D, and G developed strong peer-support relationships and became program advocates, whereas A, E, and F remained disengaged, dissatisfied, and eventually stopped participating (Figure 6). Among many reasons underlying network fragmentation, we noted a gap between the service goal and participants’ needs. That is, Colourful Youth was different from conventional programs because it aimed at enriching affective support among socially and emotionally vulnerable individuals, even those who did not acknowledge these conditions because they were struggling to regain independence and quality-of-life after undergoing trauma. In the absence of basic needs, however, some participants were desperate to find aid (e.g., unemployment benefits and job opportunities) and showed little interest in gaining affective support. In retrospect, this mismatch between the service goal and participants’ needs may have contributed to dissatisfaction and negatively influenced the group atmosphere.

#### 3.2.4. Intervention Ideas

The participants brainstormed various strategies to enhance peer support, which included (1) organizing peer-mentoring sessions, during which participants would share their concerns and needs as well as the know-how to address and fulfil them; (2) creating an online community to share useful information (e.g., about welfare services, job opportunities, and legal advice), archive their activities, and recruit new members (all of which would require as a prior condition improving digital literacy and technological accessibility among seniors); (3) teaching etiquette and manners to treat each other in the physical and virtual spaces (e.g., protecting privacy while sharing data) to prevent rude behavior during group sessions; and (4) using board/card games as tools to facilitate interaction among participants and celebrating personal events.

### 3.3. Other Findings

In both cases, most support relationships were mutual, as evidenced by their reciprocity values (Befriending = 0.9 and Colourful Youth = 0.84; reciprocity ranged from 0 to 1, and the higher the value, the more reciprocal was the group). Notably, the overall level of peer support was low, whereas the perceived peer support significantly varied among participants. In both cases, the peer-support levels ranged between ‘very little’ to ‘somewhat’ (Befriending = 1.29, Colourful Youth = 1.59), suggesting that the peer-support networks were still premature at the time of evaluation. This may be due to the relatively short duration of the service provision. It also suggests that peer support was selectively formed around the individuals with high degrees of centrality. Appendix A summarizes the network characteristics of the two cases.

### 3.4. Program Effects

To measure the programs’ effects on building peer support, we compared the changes in tie strengths between the two cases and between cliques and outliers in the two cases. We observed that cliques and outliers emerged in both cases and that people in cliques exchanged peer support, whereas the outliers felt more deprived and isolated. These comparisons would explain whether there is a difference in the perceived intensity of peer support between the clique and outlier groups and whether network fragmentation indeed influences peer support building.

We used a one-way multivariate analysis of variance (MANOVA) and univariate analysis of variance (ANOVA). The former was calculated to examine the difference in tie strengths between the datasets, and the latter to identify in which support type the difference was observed. In the comparison between the two cases, a significant difference was found (*Lambda*(3,10) = 0.433, *p* = 0.033) in affirmative support building (*F*(1,12) = 4.755, *p* = 0.050) but no significant difference was found in the other two support types. That is, participants in Befriending overall perceived a lower level of affirmative support (0.75) than those in Colourful Youth (1.79). In the comparison between the cliques and outliers, a significant difference was found (*Lambda*(3,10) = 0.450, *p* = 0.039) in affective support building (*F*(1,12) = 10.851, *p* = 0.006) but no significant difference was found in the other support types.

The result suggests that there was no significant difference in the perceived intensity of peer support between the two cases, except for in affirmative support. We interpreted, based on the interviewees’ comments, that in Befriending, affirmative support building was hindered by some members’ behavior, which invaded privacy, eroded mutual trust, and eventually divided the group. There was a significant difference in the perceived intensity of affective support between the clique and outlier groups, which suggests that participants in the cliques perceived stronger peer support than outliers and that network fragmentation influenced peer support building. This is consistent with the qualitative data that the former experienced more empathy, emotional support, and hence attachment to the program than the latter.

## 4. Discussion

### 4.1. Preconditions for Building Peer-Support Relationships

The analysis results indicated that peer support-group relationships were influenced by multiple factors, including the individual as well as sociocultural characteristics of participants, which may not have developed as practitioners had originally planned. Reflecting on our findings, we synthesized the preconditions that influenced the construction of peer-support relationships among solitary seniors. Considering that one can design for peer support programs by designing favorable conditions in which interpersonal encounters are formed and facilitated, these preconditions also imply design considerations.

#### 4.1.1. Mitigating Network Fragmentation

We observed that seniors with a high need for social and economic support were vulnerable to network fragmentation. In these cases, fragmentation within peer-support groups aggravated existing feelings of social exclusion. However, this is exactly what peer-support services are supposed to alleviate. As the Side by Side Research Consortium articulately puts it, “the sense of connection, empathy and understanding found through peer support can ease that sense of isolation and develop into a sense of community”, especially for people who do not have anyone to share their feelings with ([21], p. 30). On the other hand, fragmentation causes division and tension among group members. In this study, instances of fragmentation were associated with factors that influenced group dynamics, such as the individual, social, and cultural characteristics of participants. For instance, some seniors participating in the Befriending program perceived intergroup age variations as barriers to relationship building. We presume that this issue is associated with the hierarchical authority culture of South Korea. Other factors include the presence of spouse, education level, income, and assumptions about gender, sexuality, race, language, or religion [ibid.]. One might argue that that network fragmentation is inevitable considering the dynamism of building peer support relationships involving an exploration of who is one’s peer and who is not. Though it is natural for people to interact with others similar to themselves, the destructive nature of fragmentation in our context makes it a problem and thus it is necessary to intervene. Peer-support service coordinators thus need to consider factors that cause network fragmentation in group-building activities and seek strategies that stimulate collaboration despite these factors (e.g., involving a facilitator or identifying common interests).

#### 4.1.2. Aligning Participant Needs

It is difficult to anticipate whether peer support will be sustained among those who seek support for different reasons. The main purpose of the case-study programs was to enhance affective support among solitary seniors. However, some participants expected to obtain aid, i.e., tangible and informational support to alleviate economic hardship. When they realized this gap, some of them stopped participating and others became less engaged, thereby having a negative influence on the group atmosphere. This implies that it is necessary to align participant needs to promote network cohesion and sustain peer support. This in turn requires an understanding of participants’ motivations, needs, and expectations. From a peer-support service perspective, this means that organizers need to arrange service encounters to clearly communicate service intentions and define common participant needs prior to team building. This discrepancy of needs may be unclear until the program is fairly underway, as it was with our cases. Bowers et al. [2] advise that it is important to define the needs as they emerge, which then allows service coordinators to seek solutions to deal with them. For example, they can refresh some of the activities without changing the core value of the program [20]. In hindsight, we could have created a session or a subgroup to exchange useful information (e.g., a job search) for Colourful Youth and monitored how this experiment interacted with the overall program goal.

#### 4.1.3. Fostering Freedom and Truthfulness

Some participants admitted difficulty in being truthful and expressing themselves candidly. “I think it’s very important that we’re honest with one another. […] We still seem to be concerned about how others would look at me when I tell them about my weaknesses” (participants B and C in Colourful Youth). We also observed that relationships formed once participants began talking and formed a truthful atmosphere. In Colourful Youth, there was a point of inflection when a participant courageously decided to speak about a painful memory; this transformed the group dynamic to become more empathetic and supportive. ‘Although I never spoke with her before, I began to have good feelings and empathy for her when I heard her truthful story’ (participant C). This experience is what Side by Side Research Consortium describes as becoming oneself, and is possible when peers have a space in which they feel safe to be vulnerable and talk about difficult experiences [21]. In other words, an environment where they can be free to be themselves.

Failing to provide this environment can cause distrust among peers and impede the development of support relationships. For example, participant D in Befriending expressed concern that her in-group conversations might stigmatize her in town. Trust is a determinant of peer support [43,44]. It can be formed at the interpersonal level as illustrated by Colourful Youth, but also designed for by designing an institutional context that promotes and induces trust building, for example, by creating ground rules to protect confidentiality and emotional safety among participants [21].

#### 4.1.4. Defeating Stereotypes

It had not crossed our mind until the project ended that the organizers might have had a stereotype towards the participants—often incapable, help-seeking, and vulnerable, as this is how they were perceived socially—which may have disempowered the participants. Described as the interpersonal expectancy effect or more commonly the Pygmalion Effect, our behaviors are influenced by our perception of others and expectations of how they will behave; and the way they behave is then impacted by our expectation of them [45]. For instance, a peer support volunteer who perceives solitary seniors as passively seeking for help and thus ‘gives’ them what they need may induce attitudes and behaviors tending towards greater dependency. With hindsight, the influence of stereotypes on the cases was so subtle and unintentional as to be noticeable by the observer or the organizers. It seems plausible, however, that any attempt to recognize and deal with any stereotype is necessary for peer support organizers and participants to understand who they are and what they are capable of for themselves and others.

#### 4.1.5. Self-Identification

If the organizers of a peer support service have a stereotype about who the peers are and this stereotype is reflected in recruiting and building peer groups, it is possible that this stereotype objectifies the peers. Objectification can be disempowering, as it forces the objectified people to behave in a way that is not true to themselves. For instance, the participants may have felt that they were the subject of an experiment carried out by the organizations with power (e.g., a social welfare center or a district office that provides support to them) and may thus have been pressured—consciously or unconsciously—to behave nicely for the success of the experiment. Although objectification is not always negative and, in our context, it might be viewed as an opportunity to transform into a better self, it is still morally troublesome that they may be deprived of self-expression and self-determination (ibid.). In this regard, organizers may mitigate the effect of objectification by engaging users in self-identification of peers, i.e., understanding who oneself is and who one’s peers are, which, as Snelders argues, is a precondition to building peer support that is genuinely empowering (an email from Snelders, 18 October 2019).

### 4.2. A Relational Approach

#### 4.2.1. Process

**Figure 7:**
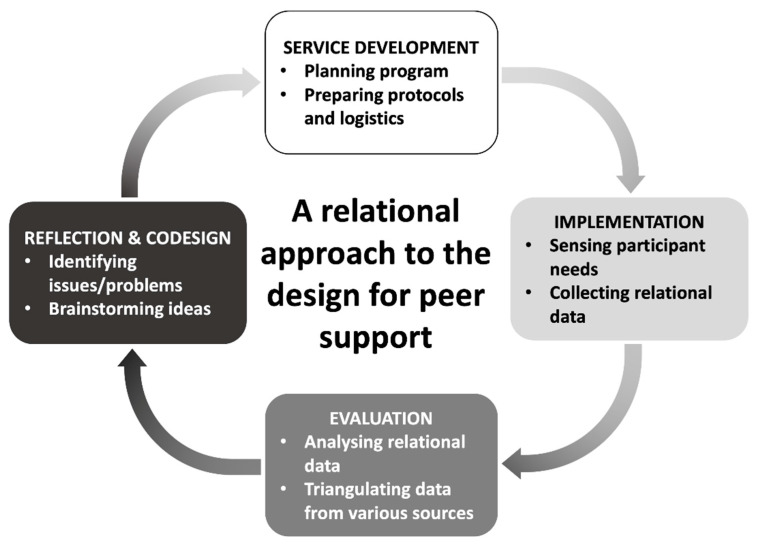
Process of designing peer-support services from a relational perspective.

Based on the lessons from the case studies, we have synthesized a relational approach to design for peer-support services. It follows an iterative process of (1) service development, (2) implementation, (3) evaluation, and (4) reflection and codesign based on the theoretical and methodological knowledge of network analysis and codesign. In the service development phase, peer-support programs are prepared, along with the protocols and logistics for implementation, which include securing resources, hiring and training staff, finding partners, and recruiting participants [22]. The protocols for data collection and analysis are also developed at this stage. During the service implementation, the program is run and the participant data are collected. The organizers remain attentive to participants’ needs and ready to intervene in the case of urgent needs. It is also at this phase that the network data are collected. Although we collected the data once, multiple collections would allow for the identification of changes in a support network. During the evaluation, participants’ relational, behavioral, and attitudinal data are analyzed and triangulated in order to draw insights. The analysis results are shared with stakeholders for reflection and codesign. In this phase, the stakeholders reflect on their implementation, identify issues/problems, and brainstorm ideas to solve these issues and improve the program. Insights gained from their feedback and ideas (e.g., preconditions for building peer-support relationships) are then fed into the next iteration (Figure 7).

#### 4.2.2. Positioning of the Approach

Existing studies for creating and maintaining peer-support services have often been developed for cohorts in specific contexts (e.g., cancers, mental illnesses, university campus). They have assumed that peer-support groups in a cohort are subject to common issues and offer a repository of tools that the leaders can adopt selectively. In this one-size-fits-most approach, there is little consideration for a process of developing new services. To the contrary, we posit that peer-support services can be better targeted to meet the relational needs of peers, and thus offer a relational approach to analyzing and designing peer-support services.

Our study reinforces the earlier works by supporting the evaluation of the front-ends of peer-support services and enabling the conception of interventions to strengthen peer support at the interpersonal level. It is useful during the planning and development phases of a service design process to assess user needs and identify design opportunities. During the evaluation phase, this can be used to assess outcomes and the impacts of interventions on peer-support networks. This relational approach employs a set of metrics that correspond to the multifaceted characteristics of a support network. The choice of which characteristics to observe depends on the purpose of the analysis and nature of the service under investigation.

## 5. Conclusions

This study introduces a relational approach to designing peer-support services and proposes a framework for analyzing peer-support networks, as well as discovering opportunities to reinforce peer support among seniors living alone. We collected network and user data through a mixed-methods approach and analyzed the network state using social network analysis, as supported by insights gained from user observations. This led to a codesign session in which service stakeholders were informed of the network state, identified relational problems, and conceptualized network-oriented interventions. Application of the framework demonstrated that it effectively supported the identification of relational problems and the conception of interventions at both the group and interpersonal levels. There are growing opportunities for practitioners to participate in developing peer-support services for and with seniors. This study supports the efficacy of such endeavors in its exploration of both theoretical and empirical knowledge.

We acknowledge that the cases in this research were relatively homogeneous because of their targeting of seniors living alone in the same cultural context. All findings thus require consideration of the related demographic and cultural limitations before interpretation or adoption in other contexts. On the other hand, our analysis of the participants’ emotions and behaviors and the insights it generated also elucidate the human attributes that transcend such limitations. For instance, feelings of empathy or jealousy, the need for social relations, and the inclination toward people similar to oneself are traits that characterize us as human beings. Another limitation is that the codesign outcomes are preliminary and limited in quantity and quality. This is because we wanted to focus on the evaluation of peer-support networks in this study and to demonstrate the potential for the relational analysis to feed into the codesign of peer-support services. We anticipate that our approach would be reinforced by integrating codesign tools that harnesses the creativity of users [42,46]—for instance, generative tools for mapping perceived peer-support relationships, tools for making desirable spaces for group activities, or tools for storytelling to describe the changes of relationships over time.

## Figures and Tables

**Table 1:** Questionnaire for peer-support network analysis (Qs 7 or 8 were repeated for Qs 1 to 6 according to respondent scores).

Data Type	Support Type		Question
**Quantitative**	Affective Support	Q1	How much does this participant make you feel liked or loved?
		Q2	How much does this participant make you feel respected or admired?
	Affirmative Support	Q3	How much can you confide in this participant?
		Q4	How much does this participant agree with or support your actions or thoughts?
	Aid	Q5	How often does this participant provide you with needed information or advice?
		Q6	If you were confined to bed for several weeks, how often could this participant visit you?
**Qualitative**		Q7	(If you scored any of Qs 1–6 between 0–1) Is there any reason or experience influencing your answer?
		Q8	(If you scored any of Qs 1–6 between 3–4) Is there any reason or experience influencing your answer?
		Q9	Which type of support do you need most from this service?
		Q10	What facilitates the construction of supportive relationships with other participants?
		Q11	What are the barriers to forming supportive relationships with other participants?
		Q12	How (if at all) can this service be improved?

**Table 2:** Metrics for measuring peer support.

Social Support Domain	Metrics	Operationalization	Application to Cases
Quantity	Network size	The number of participants in a support network	N/A
	Tie strength	The strength of interpersonal ties in a social network [39] as represented by the perceived level of (affective/affirmative/aid) support	All
Structure	Density	The proportion of support relations relative to the total possible number	All
	Reciprocity	The proportion of reciprocated support relations from the total possible number [25]	All
	Degree centrality	The extent to which an individual exchanges support with other individuals in the same network [40]	All
	Clique	How larger structures are compounded from smaller ones [37]	All
Quality	Functional content	The functional content of interpersonal relationships [31]	All

## Data Availability

Public data are not available due to privacy issues.

## Appendix A. Network Analysis Results

**Table A1:** Network characteristics of the two cases.

Case	Participant	Tie Strength			Degree Centrality		Reciprocity	Egonet Density
		Affective Support	Affirmative Support	Aid	Outdegree	Indegree		
Befriending	A	2.00	0.67	1.50	0.35	0.35	0.9	1.27
	B	1.41	0.42	1.25	0.26	0.27		1.40
	C	2.75	1.83	3.00	0.65	0.49		0.91
	D	0.58	0.50	0.92	0.17	0.21		1.52
	E	1.58	1.17	0.67	0.29	0.44		1.24
	F	2.58	1.58	0.67	0.40	0.22		1.32
	G	1.00	0.50	0.50	0.17	0.29		1.45
Colourful Youth	A	1.16	1.08	0.83	0.26	0.26	0.84	1.78
	B	3	2.25	1.67	0.58	0.52		1.35
	C	2.6	2.83	1.67	0.60	0.45		1.39
	D	1.2	1	1.33	0.28	0.50		1.78
	E	0.2	0.83	0.67	0.15	0.23		1.93
	F	1.6	2.66	1.92	0.51	0.37		1.52
	G	1.5	1.83	1.5	0.41	0.46		1.68
